# 158. Impact of Fluoroquinolone Cascade Reporting of Urine Samples on Antibiotic Prescribing Rates in a Network of Urgent Care Clinics

**DOI:** 10.1093/ofid/ofab466.158

**Published:** 2021-12-04

**Authors:** Brittani Weichman, Amanda Bushman, Rossana M Rosa

**Affiliations:** 1 UnityPoint Des Moines Iowa Lutheran Hospital, Des Moines, IA; 2 UnityPoint Health, Des Moines, IA

## Abstract

**Background:**

Cascade reporting is a type of selective reporting in which susceptibility results of certain antibiotics (either with broader spectrum or cost) are only reported if an organism is resistant to other prespecified agents. This strategy has been successfully deployed in inpatient settings but its impact in outpatient settings is less well characterized. Therefore, we aimed to evaluate the impact of cascade reporting of the antimicrobial susceptibility of fluoroquinolones on prescribing rates of select antibiotics in a network of urban Urgent Care clinics.

**Methods:**

On July 2019, the susceptibility reporting policies for urine cultures growing Enterobacterales were changed to routinely reporting a limited antibiotic panel including first and second generation cephalosporins, nitrofurantoin and trimethoprim-sulfamethoxazole (TMP-SMX), and fluroquinolone (FQ) reporting was changed to a release only in case of resistance to all agents in the limited panel. Third and fourth generation cephalosporins and carbapenems were reported only in case of resistance to all narrower spectrum agents and FQs. We then compared monthly antibiotic use in prescriptions per 1000 patient-encounters for the pre-intervention (June 2018-June 2019) and post-intervention (August 2019-December 2020) periods using an interrupted times series analysis.

**Results:**

Immediately following the change to cascade reporting, FQ prescribing decreased by 38% (incidence rate ratio [IRR] 1.38; 95% confidence interval [CI] 0.50-0.77 *p*< 0.0001) and no change in trend was subsequently seen (IRR 1.01, 95% CI 0.98-1.04; *p*=0.59) (Figure 1).Cephalexin prescribing did not immediately change following the intervention (IRR 1.20, 95% CI 1.00-1.33; *p*=0.05) but subsequently showed a trend towards increase use (IRR 1.04, 95% CI 1.02-1.06; *p*< 0.0001) (Figure 1). No immediate or trend changes in the prescribing rates of TMP-SMX or nitrofurantoin were identified (Figure 1).

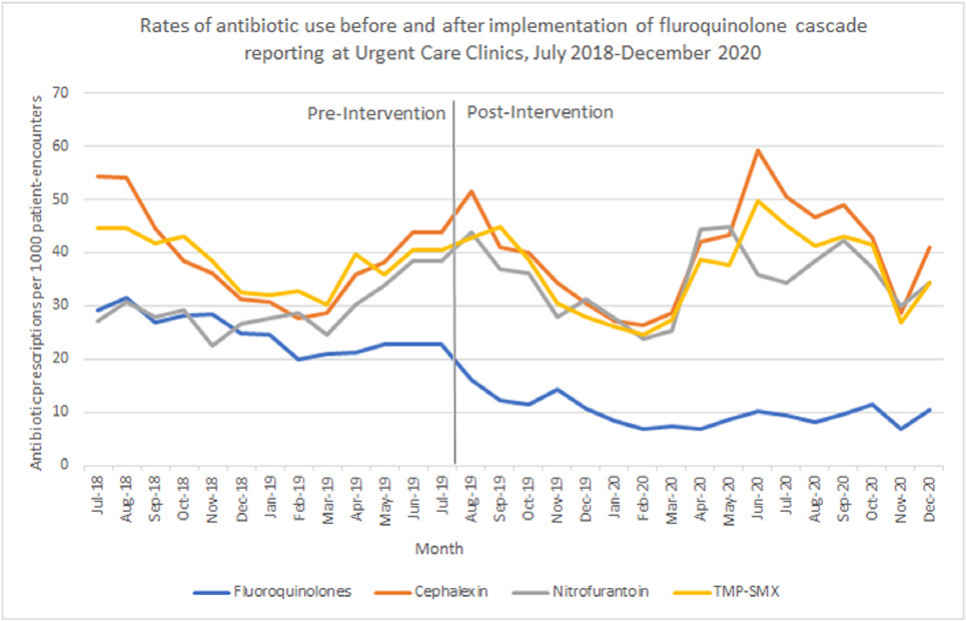

**Conclusion:**

In a network of Urgent Care clinics, cascade reporting of FQ susceptibility in urine cultures growing Enterobacterales resulted in a sustained decrease in FQ prescribing without major shifts towards prescribing of other agents. Cascade reporting should be considered as a feasible antimicrobial stewardship strategy in this outpatient setting.

**Disclosures:**

**All Authors**: No reported disclosures

